# Sericin-Induced Melanogenesis in Cultured Retinal Pigment Epithelial Cells Is Associated with Elevated Levels of Hydrogen Peroxide and Inflammatory Proteins

**DOI:** 10.3390/molecules25194395

**Published:** 2020-09-24

**Authors:** Ayyad Zartasht Khan, Catherine Joan Jackson, Tor Paaske Utheim, Sjur Reppe, Dipak Sapkota, Ole Kristoffer Olstad, Bernd Thiede, Jon Roger Eidet

**Affiliations:** 1 Institute of Clinical Medicine, Faculty of Medicine, University of Oslo, P.O. Box 1078, Blindern, 0316 Oslo, Norway; 2 Department of Medical Biochemistry, Oslo University Hospital, Kirkeveien 166, P.O. Box 4956, Nydalen, 0424 Oslo, Norway; catherinejoanjackson@gmail.com (C.J.J.); utheim2@gmail.com (T.P.U.); sjur.reppe@medisin.uio.no (S.R.); o.k.olstad@medisin.uio.no (O.K.O.); j.r.eidet@gmail.com (J.R.E.); 3 Department of Oral Biology, Faculty of Dentistry, University of Oslo, Sognsvannsveien 10, P.O. Box 1052, 0316 Oslo, Norway; dipak.sapkota@odont.uio.no; 4 Department of Plastic and Reconstructive Surgery, Oslo University Hospital, Kirkeveien 166, P.O. Box 4956, Nydalen, 0424 Oslo, Norway; 5 Department of Ophthalmology, Oslo University Hospital, Kirkeveien 166, P.O. Box 4956, Nydalen, 0424 Oslo, Norway; 6 Department of Ophthalmology, Sørlandet Hospital Arendal, P.O. Box 416, Lundsiden, 4604 Kristiansand, Norway; 7 Department of Ophthalmology, Stavanger University Hospital, P.O. Box 8100, 4068 Stavanger, Norway; 8 Lovisenberg Diakonale Hospital, Unger-Vetlesen Institute, P.O. Box 4970, Nydalen, 0440 Oslo, Norway; 9 Department of Biosciences, Faculty of Mathematics and Natural Sciences, University of Oslo, P.O. Box 1066, Blindern, 0316 Oslo, Norway; bernd.thiede@ibv.uio.no

**Keywords:** sericin, melanogenesis, superoxide dismutase, inflammation, retinal pigment epithelium, hydrogen peroxide

## Abstract

We previously demonstrated that the silk protein sericin promotes pigmentation of retinal pigment epithelium (RPE) by activating the NF-κB pathway. Among numerous agents, NF-κB can be activated by hydrogen peroxide. In the present study, we explored possible associations between reactive oxygen species and sericin-induced melanogenesis in RPE. The proteome of human fetal RPE cultured for seven days with or without 1% sericin was analyzed using ingenuity pathway analysis (IPA). The proteomic data was verified by immunofluorescence and immunoblotting. Light microscopy and scanning electron microscopy were used to assess morphology. Dihydroethidium (DHE) and dihydrorhodamine (DHR) assays were used to measure superoxide and hydrogen peroxide species. Expression levels of proteins related to inflammation, differentiation, cell survival and cell adhesion were higher in cells cultured in Dulbecco’s Modified Eagle Medium (DMEM) with 1% sericin, whereas cells cultured in DMEM alone showed higher expression levels of proteins associated with Bruch’s membrane and cytoskeleton. Despite upregulation of inflammatory proteins, sericin co-cultured RPE yielded significantly higher cell viability compared to cells cultured without sericin. Addition of sericin to culture media significantly increased hydrogen peroxide-levels without significantly affecting superoxide-levels. We suggest that sericin-induced melanogenesis in cultured RPE is associated with elevated levels of superoxide dismutase, hydrogen peroxide and inflammatory proteins.

## 1. Introduction

Research on retinal pigment epithelial (RPE) morphology and pigmentation is motivated by (1) RPE transplantation, (2) the quest to understand melanogenesis, and (3) a desire to modulate the RPE phenotype in pathological states (e.g., age-related macular degeneration (AMD)). With regards to transplantation, cutting down RPE production time, sometimes necessitating a total culture period of up to three months [1], could reduce the costs of therapy and the risk of infection associated with prolonged culture periods. Understanding RPE morphology and pigmentation could also aid in our ability to pharmacologically control cellular signaling pathways to restore a healthy RPE layer in diseases where RPE dysfunction is thought to play a key role.

Sericin is a major component of raw silk produced by *Bombyx mori* (silkworm). It has been widely investigated for potential applications in biomedicine. In 1995, Minoura et al. reported that sericin has a mitogenetic effect on murine fibroblasts [2]. Later, it was found that sericin also acts as an antioxidant [3] and promotes corneal wound healing in rats [4]. Moreover, it has been shown to display moisturizing, antiwrinkle, and antitumor properties when used in the form of lotion, cream and dietary supplement, respectively [5]. Furthermore, its application in wound dressings appeared more effective than control in treatment of split-thickness skin graft donor sites in a clinical trial [6].

We have previously reported that the addition of sericin to cell preservation medium improves RPE phenotype following a seven-day preservation period [7]. We have also shown that sericin has pro-pigmentation effects on cultured RPE, and that these effects are associated with the nuclear factor kappa-light-chain-enhancer of activated B cells (NF-κB) pathway [8].

Since sericin is considered to be a potent antioxidant [5,9], and antioxidants generally are thought to inhibit NF-κB [10,11], our previous results showing that sericin activates the NF-κB pathway [8] were unexpected. The present study was designed to follow-up on our previous transcription analysis by investigating global changes at the protein level. We herein report results of proteomic analysis of the effect of amendment of culture medium with sericin on RPE cells.

## 2. Materials and Methods

### 2.1. Materials

Primary human fetal RPE cells, complete epithelial cell medium (EpiCM), fetal bovine serum (FBS) and epithelial cell growth supplement (EpiCGS) were obtained from ScienCell Research Laboratories (San Diego, CA, USA). Dulbecco’s Modified Eagle’s Medium (high glucose, with pyruvate; hereafter named DMEM), sericin (product number: S5201), and penicillin-streptomycin were obtained from Sigma Aldrich (St Louis, MO, USA). Nunclon Δ surface 96-well plates, pipettes and other routine plastics came from VWR International (West Chester, PA, USA).

### 2.2. Cell Culture

Third passage primary human fetal RPE cells were seeded (7000 cells/cm^2^) in EpiCM consisting of 20 mL/L FBS, 10 mL/L EpiCGS, 100 U/mL penicillin and 0.1 mg/mL streptomycin (hereafter named complete EpiCM) on Nunclon Δ surface 96-well plates and cultured under routine conditions of 95% air and 5% CO_2_ at 37 °C until confluence. Thereafter, complete EpiCM was replaced by differentiation media, i.e., either: (1) DMEM or (2) DMEM with 1% (10 mg/mL) sericin. Both media contained 100 U/mL penicillin and 0.1 mg/mL streptomycin. No vehicle was used for sericin. Sterile filtered sericin powder from Sigma Aldrich was dissolved directly in DMEM (i.e., the control differentiation medium) to obtain sericin-containing differentiation medium. The differentiation media were changed every other day and the cells were maintained in differentiation media for a total of seven days.

### 2.3. In-Solution Digestion and Nano LC-Q Exactive Orbitrap Mass Spectrometry

First, cells were mechanically scraped off from culture dishes and centrifuged to make cell pellets. Then, 800 µL of SILAC phosphoprotein lysis buffer A and B (Invitrogen, Oslo, Norway) was added to make a cell slurry, which was homogenized with a pestle (20×) for mechanical breakage. An ultrasonic processor (Vibra-Cell, Sonics & Materials Inc., Newtown, CT, USA) was employed for sonication and samples were subsequently centrifuged at 16,000× *g* for 20 min at 4 °C (Centrifuge 5415R, Eppendorf, Hamburg, Germany). To 40 µL, four volumes of ice-cold acetone were added, vortexed and precipitated at −20 °C overnight. Samples were centrifuged at 16,000× *g* for 20 min at 4 °C (Centrifuge 5415R, Eppendorf, Hamburg, Germany) and the supernatant was discarded. Proteins were re-dissolved in 50 µL 6 M urea and 100 mM ammonium bicarbonate, pH 7.8. 2.5 µL of 200 mM dithiothreitol (DTT) in 100 mM Tris-HCl, pH 8 was then added for reduction and alkylation of cysteines. Following this, samples were incubated at 37°C for 1 h followed by addition of 7.5 µL 200 mM iodoacetamide for 1 h at room temperature in the dark. By adding 10 µL 200 mM DTT at 37 °C, the alkylation reaction was then quenched for 1 h. Subsequently, the proteins were digested with 10 µg trypsin for 16 h at 37 °C. We stopped the digestion by adding 5 µL 50% formic acid and the generated peptides were purified using ZipTip C18 (Millipore, Billerica, MA, USA), and dried using a concentrator (Concentrator Plus, Eppendorf, Hamburg, Germany). Nano LC-Q exactive orbitrap mass spectrometry was performed with the settings described previously [12].

### 2.4. Data Analysis

Xcalibur (version 2.5.5), Mascot generic format (*.mgf) and ProteoWizard (version 3.0.331, Palo Alto, CA, USA) were used in data analysis, as described before [12]. Peptide and protein identifications were validated using Scaffold (Proteome Software Inc., Portland, OR, USA) with the minimum probability thresholds for peptide and protein at 95.0% and 99.0%, respectively, and at least two validated peptides as specified by the Prophet peptide [13] and protein algorithms [14]. Spectral counts after normalization were used for label-free quantification as described elsewhere [15]. A two-sampled t-test in Microsoft^®^ Excel^®^ for Mac (ver. 15.19.1) was used to compare the quantity of each protein in the sericin-containing group versus the non-sericin culture group. *p*-values were considered significant if <0.05. Fold change (FC) was calculated as follows: FC = ((mean of protein expressions in RPE cultured in DMEM with 1% sericin)/(mean of protein expressions in RPE cultured in DMEM without sericin)).

Similar to other proteomic studies [16,17,18], we used QIAGEN’s Ingenuity^®^ Pathway Analysis (IPA^®^, QIAGEN Redwood City, CA, USA) to define functional patterns and analyze molecular pathways identified from the IPA library that were most significantly over-represented in the dataset by the right-tailed Fisher’s exact test. *p*-values were considered significant if <0.05.

### 2.5. Immunoblotting

Whole-cell lysates harvested with RIPA buffer and supplemented with protease inhibitor cocktail were used for Western blot experiments. Briefly, 40 µg total protein was loaded onto a 4–20% gradient gel (Criterion TGX; Bio-Rad, Hercules, CA, USA) and run at 120 V for 1 h. Protein was transferred to a PVDF membrane by wet transfer. The blots were probed with antibodies for cysteine-rich angiogenic inducer 61 protein (CYR61; 1:100; Santa Cruz sc-374129, Santa Cruz Biotechnology, Dallas, TX, USA), laminin subunit β2 (1:500; Santa Cruz sc-133241), superoxide dismutase 1 (SOD1; 1:200; Santa Cruz sc-17767), cellular retinaldehyde-binding protein (CRALBP; 1:1000; Abcam ab-154898, Abcam, Cambridge, UK), premelanosome protein (PMEL; 1:250; Santa Cruz sc-33590), and glyceraldehyde 3-phosphate dehydrogenase (GAPDH; 1:1000; Santa Cruz sc-47724). GAPDH was used as loading control. The blots were visualized with either Clarity™ Western ECL substrate (Bio-Rad) or SuperSignal™ West Femto Maximum Sensitivity Substrate (Thermo Scientific, Waltham, MA, USA). Blots were quantified in ImageJ (National Institutes of Health, Bethesda, MD, USA).

### 2.6. Light Microscopy

Three representative photomicrographs from each culture group were captured using a Leica DM microscope (Leica Microsystem, Wetzlar, Germany) and Canon EOS 5D mark II camera (Canon, Tokyo, Japan) at 200× magnification.

### 2.7. Scanning Electron Microscopy

Cells cultured on Thermanox sterile plastic coverslips (lot number 1153033, Nunc, Rochester, NY, USA), were fixed with glutaraldehyde and subsequently dehydrated in increasing ethanol concentrations and then dried according to the critical point method (Polaron E3100 Critical Point Drier, Polaron Equipment Ltd., Watford, UK) with CO_2_ as the transitional fluid. The specimens were attached to carbon stubs and coated with a 30 nm thick layer of platinum in a Polaron E5100 sputter coater before being photographed with an XL30 ESEM electron microscope (Philips, Amsterdam, The Netherlands).

### 2.8. Immunofluorescence

Cells were fixed in 100% ice-cold methanol for 15 min and subsequently washed thrice with fresh phosphate-buffered saline (PBS). Fixed cells were incubated for 45 min at room temperature in a blocking buffer consisting of 10% goat serum, 1% bovine serum albumin (BSA), 0.1% Triton X-100, 0.05% Tween-20 and 0.05% sodium azide in PBS. Cells were then incubated overnight at 4 °C with primary antibodies diluted (1:100) in blocking buffer. FITC-conjugated and Cy3-conjugated secondary antibodies were diluted (1:3000 and 1:250 respectively) in PBS with 1% BSA and incubated for one hour at room temperature. The cultures were thereafter rinsed three times in PBS and incubated with 1 μg/mL DAPI in PBS to stain cell nuclei before a final wash with PBS. Photomicrographs were captured at 200× magnification at predetermined locations in the culture wells using a Nikon Eclipse Ti fluorescence microscope with a DS-Qi1 black and white camera and a motorized stage. The exposure length and gain were maintained at a constant level for all samples and the fluorescence intensities of the FITC or Cy3 fluorochromes, which were conjugated to the secondary antibodies, were within the dynamic range of the camera. Immunofluorescence per cell was quantified by custom-made macros for ImageJ, as described previously [7,19].

### 2.9. Viability Assay

The calcein-acetoxymethyl ester (CAM) viability assay permitted quantification of calcein-stained viable cells using custom-made macros with ImageJ. CAM is a non-fluorescent compound that is hydrolyzed to calcein (strongly fluorescent) by intracellular esterases in live cells. CAM can thus be used to detect live cells. At the end of the culture period, cell medium was aspirated and cells were incubated at 37 °C for 30 min with PBS containing 1.0 μM CAM. After washing the cultures thrice with PBS, photomicrographs were captured at 200× magnification and subsequently analyzed using custom-made macros with ImageJ, as reported elsewhere [7]. In brief, unevenly transmitted light (rolling = 50) was subtracted from all 16-bit photomicrographs using the “Subtract Background”-command in ImageJ before they were converted to binary photos, Then, the culture well area covered by calcein-stained cells was automatically measured using the “Area Fraction”-command.

### 2.10. Measurement of Superoxide and Hydrogen Peroxide Reactive Oxygen Species

The probe dihydroethidium (DHE) detects intracellular mitochondrial superoxide. The cationic probe dihydrorhodamine (DHR) is readily oxidized by mitochondrial peroxyl moieties, such as hydrogen peroxide (H_2_O_2_). DHR or DHE were added at a final concentration of 3 µM to separate wells (n = 6 for each probe). After incubation for 90 min at 24 °C ambient CO_2_ (DHR), or for 20 min at 32 °C ambient CO_2_ (DHE), cells were detached with 3 min trypsin incubation, washed and resuspended in ice-cold HBSS + 4% FBS. Samples were kept on ice and analyzed using a BD Accuri C6 flow cytometer. The probes were transformed from non-fluorescent to fluorescent compounds by oxidative burst intermediates within the cell. Excitation of the DHR dye at 488 nm produced green fluorescence with a peak at 530 nm detected using the standard filter 530/30 (FL1). Excitation of the DHE dye at 488 nm produced red fluorescence with a peak at 600 nm detected using the standard filter 585/40 nm (FL2). The mean fluorescence of each sample was recorded. Samples without addition of probe (probe negative control +/− 1% sericin) were included. The Mann-Whitney U-test was used to compare two groups. A two-tailed *p*-value < 0.05 was considered significant.

### 2.11. Quantification of Pigmentation

Cellular pigment was measured using ImageJ in light microscopy images of RPE cultured with or without sericin. In brief, 200× photomicrographs were converted to 8-bit images before employing the “Subtract Background”-command in ImageJ. Based on pixel whiteness, a threshold was then applied to quantify the dark area fraction (%) of the images that represented cellular pigment. The same macro was used on all photomicrographs. A two-sample two-tailed t-test was used to compare the sericin-supplemented and control groups. A *p*-value < 0.05 was considered significant.

### 2.12. Quantification of Microvilli

Three investigators independently counted the number of microvilli in 10 µm × 10 µm sections of SEM micrographs from each culture group (n = 3) which were obtained at predetermined locations. All three investigators were blinded to the origins of the micrographs. Means were calculated based on the counts from the investigators. A two-sample, two-tailed t-test was used to compare the sericin-supplemented and control groups. A *p*-value < 0.05 was considered significant.

## 3. Results

### 3.1. Effects of Sericin on Protein Expression

**Figure 1:**
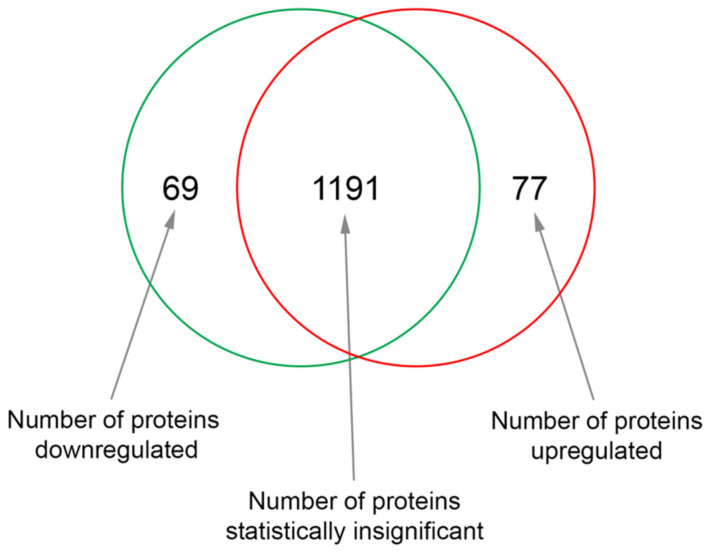
The Venn diagram shows the number of proteins that were significantly upregulated or downregulated in human fetal retinal pigment epithelial cells (RPE) cultured in Dulbecco’s Modified Eagle’s Medium (DMEM) with 1% sericin compared to cells cultured in DMEM without sericin.

**Figure 2:**
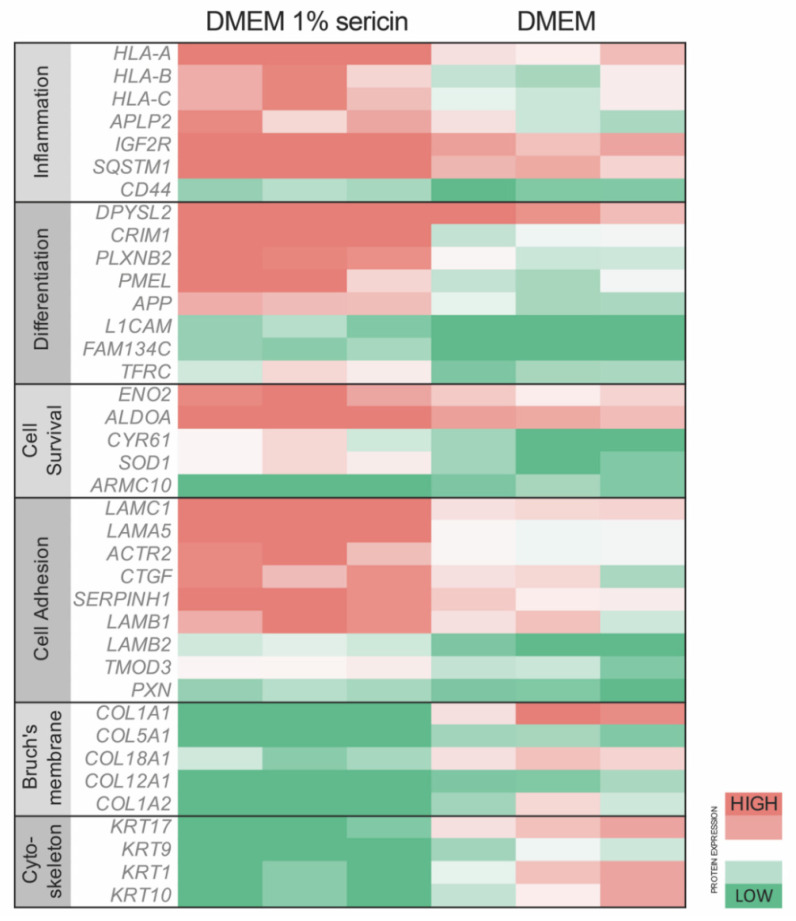
Primary human fetal retinal pigment epithelial cells (RPE) were cultured in Dulbecco’s Modified Eagle’s Medium (DMEM) with or without 1% sericin. All cultures were subjected to proteomic analysis to evaluate differences in protein expression. Relative protein expression of key proteins related to inflammation, differentiation, cell survival and cell adhesion was higher in cells cultured in DMEM with 1% sericin, whereas cells cultured in DMEM without sericin showed higher expression levels of proteins associated with Bruch’s membrane and cytoskeleton. *N* = 3.

To investigate how exposure to sericin affects RPE in vitro, we analyzed the proteomic profile of RPE cultured in DMEM with 1% sericin in comparison to RPE cultured in DMEM alone. The quantitative MS/MS analysis identified 1337 proteins with two peptides or more at 95.0% minimum peptide threshold and 99.0% minimum protein threshold. Of these, 77 proteins were significantly (*p* < 0.05) upregulated (Table 1), while 69 proteins were significantly (*p* < 0.05) downregulated (Table 2) in RPE cultured in DMEM with 1% sericin compared to cells cultured in DMEM alone (Figure 1). To explore the functions of these up- and downregulated proteins, we searched each protein in the UniProt [20] and GeneCards [21] protein databases. The majority of the differentially expressed proteins were related to inflammation, RPE differentiation, cell survival, cell adhesion, Bruch’s membrane and cytoskeleton. Within these functions, a clear separation of protein expression direction was observed between the two culture groups (Figure 2). The quantitative MS/MS analysis thus indicated that in vitro sericin exposure significantly alters protein expression in RPE.

### 3.2. Immunofluorescence and Immunoblotting

**Figure 3:**
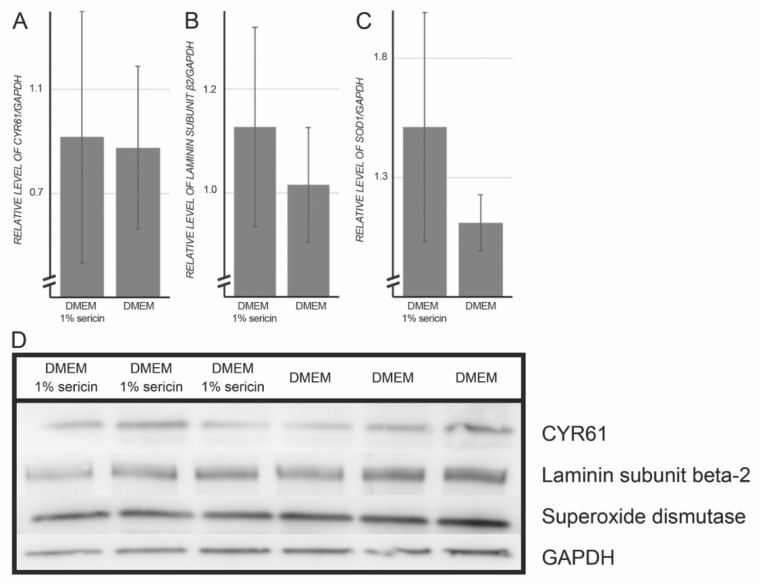
Whole-cell lysates of human retinal pigment epithelial cells (RPE) cultured for seven days in Dulbecco’s Modified Eagle’s Medium (DMEM) with or without 1% sericin were subjected to Western blot experiments. Glyceraldehyde 3-phosphate dehydrogenase (GAPDH) was used as loading control. Fold change (FC) was calculated as follows: FC = ((mean of protein expressions in RPE cultured in DMEM with 1% sericin)/(mean of protein expressions in RPE cultured in DMEM without sericin)). (**A**) Cysteine-rich angiogenic inducer 61 (CYR61); FC: 1.05; *p*-value: 0.90. (**B**); Laminin subunit β-2; FC: 1.11; *p*-value: 0.43. (**C**) Superoxide dismutase; FC: 1.36; *p*-value: 0.23. (**D**) Western blot. Error bars represent standard deviation of the mean. *N* = 3.

To validate the MS/MS proteomic results, the expression levels of CYR61, laminin subunit β-2 and superoxide dismutase were assessed by immunofluorescence and immunoblotting in both culture groups. The rationale behind selecting targets to validate by Western blot was to verify relevant biological aspects of the proteomic dataset, as recommended elsewhere [22], instead of just validating the most abundant proteins. Because the proteomic dataset indicated that proteins related to inflammation, differentiation, cell adhesion and superoxide dismutase were upregulated in the sericin-treated group, targets to be validated by immunoblotting were chosen to represent some of these biological functions. Immunofluorescence and Western blot experiments confirmed the presence of the selected targets (Table 3 & Figure 3).

### 3.3. Sericin Increases Viability Despite Inducing an Inflammatory Response

**Figure 4:**
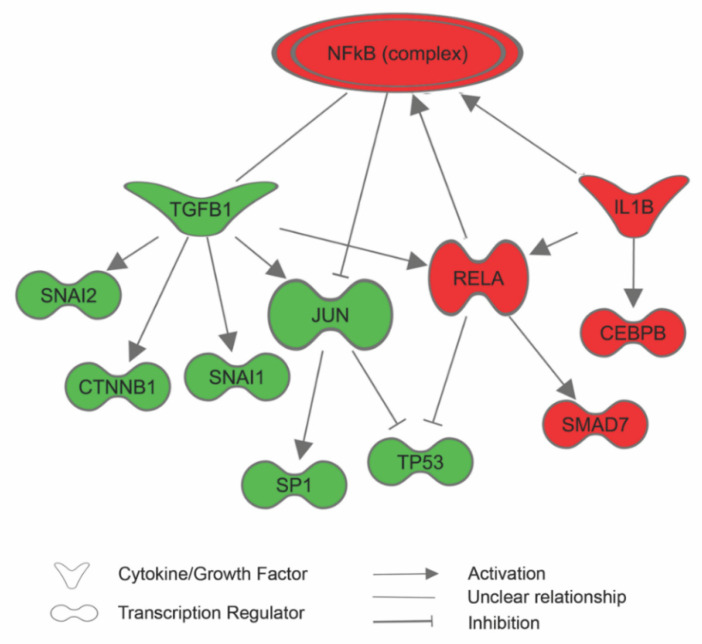
Based on the differentially expressed proteins, Ingenuity Pathway Analysis (IPA) predicted that the NF-κB pathway was activated in human fetal retinal pigment epithelial cells (RPE) cultured in Dulbecco’s Modified Eagle’s Medium (DMEM) with 1% sericin. The prediction was based on upregulation (red symbols) of several of its promoters and the concurrent downregulation (green symbols) of regulators that are known to be inhibited by the NF-κB pathway. For the sake of clarity, some extraneous relationships have been omitted, but are included in the Supplementary Material.

**Figure 5:**
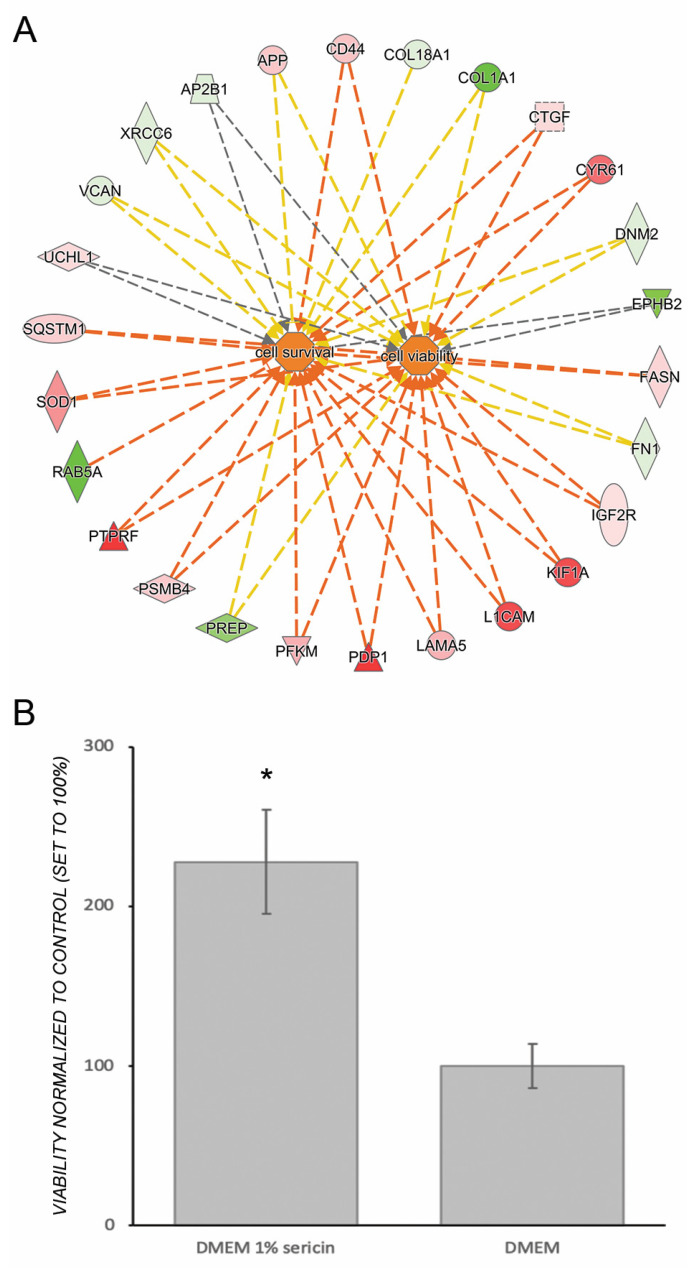
Ingenuity Pathway Analysis (IPA) predicted a downstream effect compatible with increased viability and survival of human fetal retinal pigment epithelial cells (RPE) cultured in in Dulbecco’s Modified Eagle’s Medium (DMEM) with 1% sericin compared to cells cultured without sericin (**A**). Red and pink symbols indicate increased protein levels upon culturing in presence of sericin, while green symbols indicate reduced protein levels. The dotted lines indicate indirect relationships leading to activation (orange). Yellow and grey dotted lines indicate inconsistent relationships and no predicted effects, respectively. To verify the effects on cell survival and viability predicted by IPA, the culture well area of CAM-stained live cells was measured in confluent layers of RPE cultured in DMEM with 1% sericin or DMEM alone. The bar chart (**B**) shows culture well area covered by live cells in RPE cultured in DMEM with 1% sericin normalized to the control cultures, i.e., RPE cultured in DMEM alone (100%). RPE cultured in DMEM with 1% sericin yielded significantly higher cell viability compared to cells cultured without sericin (*p* = 0.04). Error bars represent standard deviation of the mean. * *p* < 0.05. *N* = 3.

The proteomic data revealed that RPE cultured for seven days in DMEM with 1% sericin expressed significantly higher levels of inflammation-related proteins in comparison to RPE cultured in DMEM only (Figure 2). IPA predicted that the NF-κB pathway, important in pro-inflammatory signaling [23], was upregulated in RPE cultured in DMEM with 1% sericin (*p* = 0.001). The prediction was based on upregulation of several of its promoters and the concurrent downregulation of regulators that are known to be inhibited by NF-κB (Figure 4). Despite upregulation of inflammatory proteins, IPA suggested a downstream effect compatible with increased cell viability and survival in the sericin-supplemented group (Figure 5A). To confirm this finding, we performed a CAM-based viability assay. CAM is a non-fluorescent compound that is hydrolyzed to calcein (strongly fluorescent) by intracellular esterases in live cells. CAM can thus be used to detect live cells. We measured the culture well area covered by CAM-stained live RPE cells in both culture groups. RPE cultured in DMEM with 1% sericin yielded significantly higher cell viability compared to cells cultured without sericin (*p* = 0.04) (Figure 5B). These results show that sericin increases viability despite initiating an inflammatory response in RPE in vitro.

### 3.4. Sericin Promotes Pigmentation of Cultured Human Retinal Pigment Epithelial Cells

**Figure 6:**
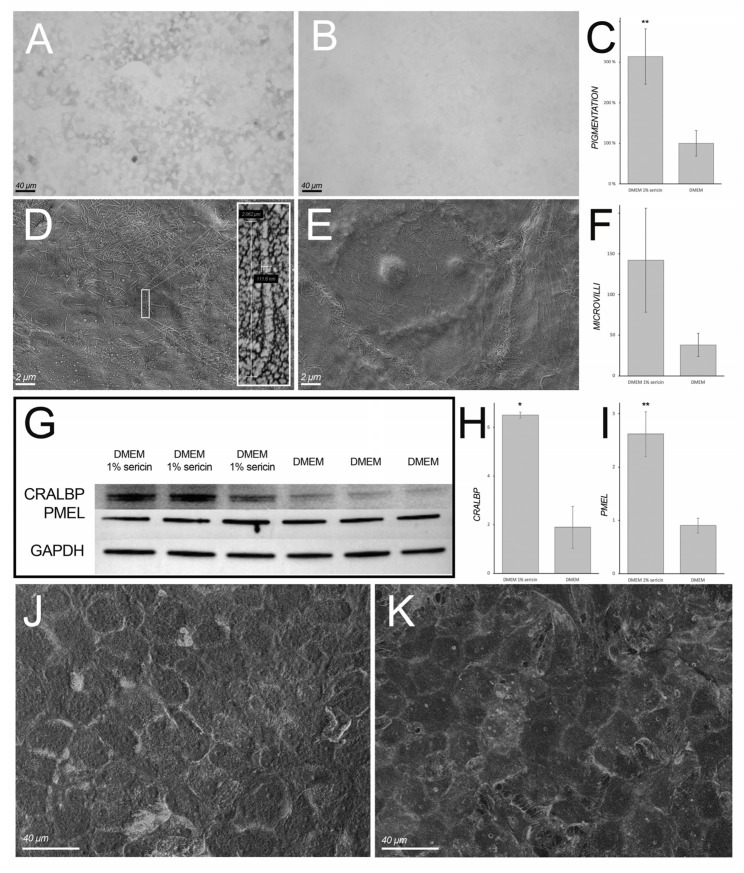
Light microscopy images (grayscale) of RPE cultured in Dulbecco’s Modified Eagle’s Medium (DMEM) with (**A**) or without (**B**) 1% sericin in which the former group contained significantly more pigmented cells. The bar chart (**C**) shows culture well area covered by pigmented cells normalized to control cultures (RPE cultured in DMEM only; set to 100%). In RPE cultured in DMEM with 1% sericin (**D**), apical microvilli appeared to be more abundant in comparison to cells cultured in DMEM only (**E**). However, the difference was not statistically significant (F). The bar chart shows mean number of microvilli in 10 µm × 10 µm sections of SEM micrographs counted independently by three blinded investigators (**F**). To assess RPE-specific maturation markers, immunoblotting (**G**) was used to quantify cellular retinaldehyde-binding protein (CRALBP) and premelanosome protein (PMEL), which were both most abundant in the sericin-supplemented cultures. Glyceraldehyde 3-phosphate dehydrogenase (GAPDH) was used as loading control. The bar charts show relative expression of CRALBP/GAPDH (**H**) and relative expression of PMEL/GAPDH (**I**). Tightly adjoined, hexagonal cells with typical cobblestone morphology were observed in both groups (**J**: DMEM with 1% sericin; **K**: DMEM only). All bar charts (**C**,**F**,**H**,**I**) show mean values ± standard deviation error bars. * *p* < 0.05. ** *p* < 0.001.

Light microscopy and scanning electron microscopy (SEM) were employed to assess sericin’s effect on the morphology of cultured RPE. Light microscopy showed pigmented RPE in sericin-supplemented cultures (Figure 6A), whereas cells cultured in only DMEM showed markedly less pigmentation (Figure 6B). Quantification of the dark area fraction in light microscopy images revealed significantly higher pigmented culture well area in the sericin-supplemented group (314% ± 68%; *p* < 0.001) in comparison to cultures not supplemented with sericin (set to 100%) (Figure 6C). SEM showed tightly adjoined hexagonal cells with typical cobblestone morphology in both culture groups (Figure 6J,K). However, apical microvilli appeared to be more abundantly present in cells cultured in DMEM with 1% sericin (Figure 6D) compared to DMEM only (Figure 6E). Quantification of microvilli, albeit not significant, supported this observation (microvilli count in DMEM with 1% sericin: 142 ± 64; microvilli count in DMEM only: 38 ± 14; *p* = 0.051) (Figure 6F). To assess the quantity of RPE-specific maturation markers CRALBP and PMEL, immunoblotting was employed (Figure 6G). CRALBP and PMEL were both most abundant in RPE cultured in DMEM with 1% sericin (344% ± 14%; *p* = 0.01; Figure 6H, and 290% ± 47%; *p* < 0.001; Figure 6I, respectively) compared to cells cultured in DMEM only (set to 100%). Collectively, these findings suggest that sericin-supplementation promotes the pigmentation and maintains the epithelial morphology of RPE in vitro.

### 3.5. Effects of Sericin on Superoxide Dismutase Substrate and Product

**Figure 7:**
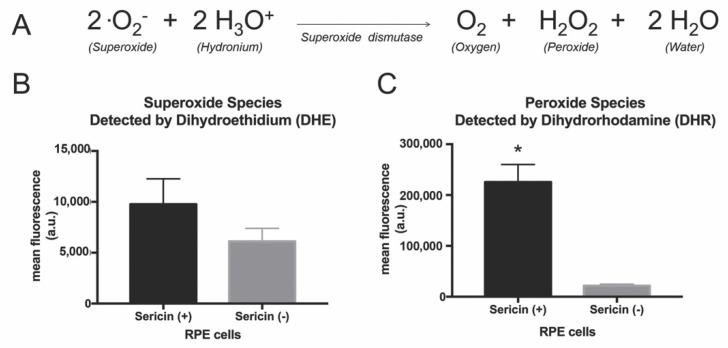
Superoxide dismutase is an antioxidant enzyme that catalyzes the dismutation of superoxide into oxygen, water and hydrogen peroxide (**A**). Proteomic analysis revealed that this enzyme was increased 5.4-fold in cells cultured with sericin compared to cells cultured without sericin. We performed dihydroethidium (DHE) and dihydrorhodamine (DHR) assays to study the levels of the key substrate and product of this enzyme. The DHE assay measures superoxide, while the DHR assay quantifies hydrogen peroxide. The DHE assay revealed no significant increase in superoxide in the sericin-containing RPE cultures compared to control cultures (**B**). The DHR assay showed significantly higher levels of hydrogen peroxide in RPE cultured with sericin (**C**). All bar charts show mean values ± standard deviation error bars. * *p* < 0.05.

IPA revealed higher levels of superoxide dismutase, an enzyme that catalyzes the dismutation of superoxide into oxygen, water and hydrogen peroxide [24], in RPE cultured with sericin in comparison to RPE cultured without sericin. Quantification of superoxide (one of the substrates of superoxide dismutase) with a dihydroethidium (DHE) assay revealed no significant increase in superoxide in the sericin-containing RPE cultures compared to control cultures (*p* = 0.052; Figure 7B). A dihydrorhodamine (DHR) assay showed significantly higher levels of hydrogen peroxide (one of the products of the superoxide dismutase enzyme) in RPE cultured with sericin (10.5-fold increase, compared to the control where no sericin was added; *p* = 0.002; Figure 7C).

## 4. Discussion

In the current study, we investigated how sericin affects the proteome of primary human fetal RPE cells. Following a seven-day culture period, we found that cultures supplemented with sericin showed higher levels of proteins associated with inflammation, differentiation, cell survival and cell adhesion. The sericin-treated cultures in the present experiments showed better pigmentation at the end of the culture period. Furthermore, IPA analysis of the proteomic dataset predicted that the NF-κB pathway, important in pro-inflammatory signaling, was upregulated in RPE cultured in DMEM with 1% sericin. This is in agreement with a previous transcription analysis report by our group in which we demonstrated that sericin conduces pigmentation of RPE by activating the NF-κB pathway [8]. Despite the higher levels of inflammation-associated proteins, and the predicted activation of the NF-κB pathway in sericin-supplemented RPE cultures, significantly higher cell viability was observed in these cultures compared to cells cultured without sericin.

Analysis of the proteome of cultured RPE revealed higher levels of inflammatory proteins and superoxide dismutase in the sericin-supplemented cultures. These findings are supported by reports proposing sericin-associated superoxide dismutase activity [25,26]. Moreover, through Affymetrix microarray analysis, we recently reported that sericin upregulates superoxide dismutase genes in RPE cultured with sericin [8]. Superoxide dismutase is an enzyme that catalyzes the dismutation of superoxide into oxygen, water and hydrogen peroxide. Since NF-κB can be activated by hydrogen peroxide [27], we wished to investigate whether sericin promotes activation of the NF-κB pathway through regulating the level of this reactive oxygen species. A DHR assay showed a significantly higher level of hydrogen peroxide (one of the products of the superoxide dismutase enzyme) in RPE cultured with sericin. However, because superoxide dismutase is an enzyme, its elevated levels in sericin-supplemented cultures (as shown by the proteomic and western blot experiments), and the elevated levels of its product (as shown by the DHR assay), could also be caused by an increased amount of its substrates (superoxide and hydronium). This would fit with the proteomic data indicating a sericin-induced inflammatory reaction, and also the fact that sericin is a xenobiotic that, in theory, could cause ROS-generation through activation of inflammatory processes. However, quantification of superoxide (one of the substrates of superoxide dismutase) with a DHE assay revealed no significant increase in superoxide in the sericin-containing RPE cultures compared to control cultures. Even though the DHE assay did not show significantly elevated levels of superoxide, we cannot rule out that an increased catalytic activity of superoxide dismutase is responsible for this finding. Hence, the data presented herein cannot be used to conclude whether the addition of sericin causes higher levels of superoxide, which in turn increase the activity of the superoxide dismutase enzyme, or if the increased levels of superoxide dismutase are directly induced by sericin.

The DHR assay showed a significantly higher level of hydrogen peroxide in RPE cultured with sericin. Interestingly, hydrogen peroxide has been reported to activate NF-κB [27], and we have shown that increased NF-κB is associated with increased pigmentation in cultured RPE [8]. In epidermal cells, generation of hydrogen peroxide through ultraviolet radiation is known to cause melanogenesis [28]. Furthermore, it has been shown that hydrogen peroxide increases melanin synthesis and pigmentation in primary human melanocytes and keratinocytes [29]. Recently, Kim et al. reported that temporary exposure of melanoma cells to hydrogen peroxide can induce melanogenesis by increasing ATP, intracellular cAMP and melanogenesis-related genes [30]. Thus, we hypothesize that the pro-pigmentation effects of sericin in RPE in vitro are associated with inflammation and increased levels of hydrogen peroxide. However, significantly higher RPE cell viability with sericin addition suggests that hydrogen peroxide is maintained at a manageable level. Indeed, Tang et al. showed that a low level of hydrogen peroxide resulted in melanin synthesis in melanocytes, while high levels decreased melanin synthesis and increased cell death [29]. Our proteomic results indicate that hydroxyacylglutathione hydrolase (HAGH) was significantly increased (Table 1) in sericin-supplemented cells. HAGH is a thiolesterase enzyme that produces reduced glutathione and D-lactate, which is used in the antioxidant reaction converting hydrogen peroxide to water and oxygen, combatting oxidative stress. Thus, increased antioxidant activity by HAGH may be implicated in maintenance of hydrogen peroxide at a balanced level that promotes RPE pigmentation and maintains viability.

In a previous study on preservation of cultured primary human fetal RPE, we showed that the fraction of pigmented cells was inversely related to storage temperature [7]. The precise mechanisms behind these results were not fully understood. However, in light of the current investigation suggesting a role of superoxide dismutase and hydrogen peroxide in melanogenesis, the reported findings can be explained based on the temperature dependence of superoxide dismutase activity. Contrary to classic temperature-dependent enzyme activity, superoxide dismutase activity increases with decreasing temperature [31]. Hence, if superoxide dismutase plays a central role in melanogenesis in RPE, an increase in temperature would decrease superoxide dismutase activity, lower hydrogen peroxide-levels and thus decrease melanogenesis.

Accompanying the upregulation of differentiation-related proteins in sericin-supplemented RPE cultures (in comparison to cells cultured in DMEM only) is an increase of proteins associated with cell adhesion (Figure 2). This observation is particularly interesting in the light of a report that showed that cell adhesion was important for RPE melanogenesis, suggesting an interrelationship between RPE cell-to-cell adhesion and pigmentation [32]. The same study noted that the process of melanogenesis was not evenly distributed in the RPE cultures. Rather, it apparently started in scattered cells, subsequently spreading to neighboring cells. This phenomenon was also observed by us during the present experiments and is evident when studying the photomicrograph in Figure 6A. Aside from underscoring the importance of cell-to-cell adhesion, this interesting phenomenon suggests that RPE cells may stimulate adjacent cells through intercellular junctions and/or paracrine mediators. It also serves to establish a relationship between the upregulated protein groups presented in this study.

In conclusion, we have demonstrated that sericin-induced melanogenesis in cultured fetal RPE is associated with elevated levels of superoxide dismutase, hydrogen peroxide and inflammatory proteins. In the future, it could be interesting to study whether the effects of sericin reported herein can be replicated in other cells such as melanocytes, keratinocytes, iris pigment epithelial cells or induced pluripotent stem cell-derived RPE. If so, new avenues for treating pigmentation disorders may unfold, and the time-consuming differentiation of induced pluripotent stem cell-derived RPE from pluripotent stem cells could be shortened, thereby reducing a significant hurdle in regenerative medicine strategies for blinding diseases (e.g., AMD).

## Figures and Tables

**Table 1:** Significantly upregulated proteins in human fetal retinal pigment epithelial cells (RPE) cultured in Dulbecco’s Modified Eagle’s Medium (DMEM) with 1% sericin compared to cells cultured in DMEM without sericin, sorted by decreasing fold change (FC). FC was calculated as follows: FC = ((mean of protein expressions in RPE cultured in DMEM with 1% sericin)/(mean of protein expressions in RPE cultured in DMEM without sericin)). *N* = 3. FC: Fold change.

Protein Name	Gene Symbol	FC	*p*-Value
Hydroxyacylglutathione hydrolase, mitochondrial	*HAGH*	15.376	0.039
Low-density lipoprotein receptor	*LDLR*	13.523	0.003
(Pyruvate dehydrogenase (acetyl-transferring))-phosphatase 1, mitochondrial	*PDP1*	12.853	0.010
Receptor-type tyrosine-protein phosphatase F	*PTPRF*	11.652	0.030
Laminin subunit β-2	*LAMB2*	10.453	0.001
Translational activator GCN1	*GCN1L1*	9.168	0.003
Kinesin-like protein KIF1A	*KIF1A*	9.129	0.029
Neural cell adhesion molecule L1	*L1CAM*	9.129	0.029
Protein FAM134C	*FAM134C*	8.789	0.009
Pyridoxine-5′-phosphate oxidase	*PNPO*	8.789	0.009
Hsc70-interacting protein	*ST13*	7.696	0.039
Protein CYR61	*CYR61*	7.346	0.020
Isocitrate dehydrogenase (NAD) subunit α, mitochondrial	*IDH3A*	6.797	0.047
Leucine-rich repeat neuronal protein 1	*LRRN1*	5.691	0.029
Glycine cleavage system H protein, mitochondrial	*GCSH*	5.495	0.020
Protein arginine *N*-methyltransferase 5	*PRMT5*	5.495	0.020
Superoxide dismutase (Cu-Zn)	*SOD1*	5.443	0.003
Seizure 6-like protein 2	*SEZ6L2*	4.820	0.002
Tetraspanin-10	*TSPAN10*	4.669	0.040
Sortilin	*SORT1*	4.492	0.041
Cysteine-rich motor neuron 1 protein	*CRIM1*	4.437	0.009
Sodium-coupled neutral amino acid transporter 2	*SLC38A2*	4.106	0.021
6-phosphofructokinase, muscle type	*PFKM*	3.903	0.015
Laminin subunit α-5	*LAMA5*	3.788	0.011
Tubulin--tyrosine ligase-like protein 12	*TTLL12*	3.621	0.035
Melanocyte protein PMEL	*PMEL*	3.123	0.021
Paxillin	*PXN*	3.097	0.029
3-hydroxyisobutyrate dehydrogenase, mitochondrial	*HIBADH*	3.056	0.030
Plexin-B2	*PLXNB2*	3.049	0.011
40S ribosomal protein S28	*RPS28*	2.956	0.034
CD44 antigen	*CD44*	2.956	0.034
Cullin-associated NEDD8-dissociated protein 1	*CAND1*	2.951	0.049
Transferrin receptor protein 1	*TFRC*	2.847	0.029
Amyloid β A4 protein	*APP*	2.838	0.001
Calpain-1 catalytic subunit	*CAPN1*	2.751	0.026
60S ribosomal protein L18a	*RPL18A*	2.704	0.007
Proteasome subunit β type-4	*PSMB4*	2.692	0.026
7-dehydrocholesterol reductase	*DHCR7*	2.600	0.048
60S ribosomal protein L13	*RPL13*	2.551	0.009
Small nuclear ribonucleoprotein G	*SNRPG*	2.551	0.009
NADH dehydrogenase (ubiquinone) iron-sulfur protein 2, mitochondrial	*NDUFS2*	2.546	0.018
Sequestosome-1	*SQSTM1*	2.487	0.001
Heterogeneous nuclear ribonucleoprotein A1	*HNRNPA1*	2.416	0.023
HLA class I histocompatibility antigen, B-8 α chain	*HLA-B*	2.365	0.031
Laminin subunit γ-1	*LAMC1*	2.334	0.006
Eukaryotic translation initiation factor 4H	*EIF4H*	2.252	0.019
Rab GDP dissociation inhibitor β	*GDI2*	2.245	0.028
Actin-related protein 2	*ACTR2*	2.228	0.022
Amyloid-like protein 2	*APLP2*	2.203	0.042
GTP-binding nuclear protein Ran	*RAN*	2.202	0.009
Syntenin-1	*SDCBP*	2.173	0.006
Integrin α-3	*ITGA3*	2.142	0.027
Tropomodulin-3	*TMOD3*	2.131	0.016
HLA class I histocompatibility antigen, Cw-12 α chain	*HLA-C*	2.065	0.012
Fatty acid synthase	*FASN*	2.050	0.025
HLA class I histocompatibility antigen, A-30 α chain	*HLA-A*	2.014	0.006
Guanine nucleotide-binding protein G(s) subunit α isoforms XLas	*GNAS*	1.995	0.010
Catechol O-methyltransferase	*COMT*	1.899	0.045
Connective tissue growth factor	*CTGF*	1.881	0.049
Ubiquitin carboxyl-terminal hydrolase isozyme L1	*UCHL1*	1.861	0.036
Serpin H1	*SERPINH1*	1.851	0.003
Thioredoxin reductase 1, cytoplasmic	*TXNRD1*	1.807	0.049
Pyruvate dehydrogenase E1 component subunit β, mitochondrial	*PDHB*	1.784	0.004
WD repeat-containing protein 1	*WDR1*	1.776	0.009
Laminin subunit β-1	*LAMB1*	1.768	0.049
Importin subunit β-1	*KPNB1*	1.763	0.048
Ribonuclease inhibitor	*RNH1*	1.744	0.023
T-complex protein 1 subunit eta	*CCT7*	1.680	0.011
Fructose-bisphosphate aldolase A	*ALDOA*	1.643	0.023
γ-enolase	*ENO2*	1.598	0.017
Cation-independent mannose-6-phosphate receptor	*IGF2R*	1.575	0.009
S-formylglutathione hydrolase	*ESD*	1.573	0.025
14-3-3 protein β/α	*YWHAB*	1.515	0.037
Heat shock protein HSP 90-α	*HSP90AA1*	1.460	0.025
Dihydropyrimidinase-related protein 2	*DPYSL2*	1.452	0.033
Myoferlin	*MYOF*	1.349	0.044
Coronin-1C	*CORO1C*	1.235	0.030

**Table 2:** Significantly downregulated proteins in human fetal retinal pigment epithelial cells (RPE) cultured in Dulbecco’s Modified Eagle’s Medium (DMEM) with 1% sericin compared to cells cultured in DMEM without sericin. Fold change (FC) was calculated as follows: FC = ((mean of protein expressions in RPE cultured in DMEM with 1% sericin)/(mean of protein expressions in RPE cultured in DMEM without sericin)). *N* = 3. FC: Fold change.

Protein Name	Gene Symbol	FC	*p*-Value
Ras-related protein Rab-5A	*RAB5A*	0.000	0.047
Collagen α-2(I) chain	*COL1A2*	0.000	0.041
Myosin phosphatase Rho-interacting protein	*MPRIP*	0.000	0.024
Collagen α-1(XII) chain	*COL12A1*	0.000	0.021
Fibulin-1	*FBLN1*	0.000	0.021
Lamina-associated polypeptide 2, isoforms β/γ	*TMPO*	0.000	0.021
Zinc finger protein ubi-d4	*DPF2*	0.000	0.021
Lysyl oxidase homolog 1	*LOXL1*	0.000	0.019
Armadillo repeat-containing protein 10	*ARMC10*	0.000	0.018
Heterogeneous nuclear ribonucleoprotein D-like	*HNRNPDL*	0.000	0.018
Tetraspanin-6	*TSPAN6*	0.000	0.018
Collagen α-1(I) chain	*COL1A1*	0.000	0.012
Cysteine-rich with EGF-like domain protein 1	*CRELD1*	0.000	0.010
Fragile X mental retardation syndrome-related protein 2	*FXR2*	0.000	0.010
Prolyl endopeptidase	*PREP*	0.000	0.010
Ras-related protein Rab-13	*RAB13*	0.000	0.010
RNA-binding protein 42	*RBM42*	0.000	0.010
Keratin, type I cytoskeletal 9	*KRT9*	0.000	0.008
Ephrin type-B receptor 2	*EPHB2*	0.000	0.007
Single-stranded DNA-binding protein, mitochondrial	*SSBP1*	0.000	0.007
Transformer-2 protein homolog α	*TRA2A*	0.000	0.007
Collagen α-1(V) chain	*COL5A1*	0.000	0.006
Hydroxyacyl-coenzyme A dehydrogenase, mitochondrial	*HADH*	0.000	0.006
Prolyl 4-hydroxylase subunit α-1	*P4HA1*	0.000	0.004
Keratin, type I cytoskeletal 17	*KRT17*	0.049	0.001
Keratin, type II cytoskeletal 1	*KRT1*	0.063	0.013
Keratin, type I cytoskeletal 10	*KRT10*	0.075	0.038
Septin-11	*SEPT11*	0.114	0.002
AP-2 complex subunit β	*AP2B1*	0.121	0.043
Prolyl 3-hydroxylase 3	*LEPREL2*	0.136	0.021
Putative tropomyosin α-3 chain-like protein	*TPM3L*	0.146	0.018
Serine/arginine-rich splicing factor 1	*SRSF1*	0.173	0.026
Septin-7	*SEPT7*	0.174	0.038
S-phase kinase-associated protein 1	*SKP1*	0.207	0.047
Myristoylated alanine-rich C-kinase substrate	*MARCKS*	0.217	0.020
Dynactin subunit 1	*DCTN1*	0.228	0.039
Epiplakin	*EPPK1*	0.250	0.015
Versican core protein	*VCAN*	0.259	0.016
EGF-containing fibulin-like extracellular matrix protein 1	*EFEMP1*	0.271	0.016
Y-box-binding protein 3	*YBX3*	0.294	0.026
Basement membrane-specific heparan sulfate proteoglycan core protein	*HSPG2*	0.300	0.004
Myosin regulatory light polypeptide 9	*MYL9*	0.331	0.032
Collagen α-1(XVIII) chain	*COL18A1*	0.338	0.004
Fibronectin	*FN1*	0.365	0.007
U1 small nuclear ribonucleoprotein 70 kDa	*SNRNP70*	0.374	0.031
Fibrillin-2	*FBN2*	0.390	0.001
X-ray repair cross-complementing protein 6	*XRCC6*	0.397	0.021
Fibulin-2	*FBLN2*	0.398	0.005
Fibrillin-1	*FBN1*	0.419	0.001
Histone H4	*HIST1H4A*	0.442	0.007
β-galactosidase	*GLB1*	0.452	0.026
Nodal modulator 3	*NOMO3*	0.456	0.021
Drebrin	*DBN1*	0.472	0.037
Histone H3.1	*HIST1H3A*	0.481	0.015
Calmodulin	*CALM1*	0.487	0.022
General transcription factor II-I	*GTF2I*	0.551	0.028
Myosin regulatory light chain 12B	*MYL12B*	0.563	0.001
Spectrin α chain, nonerythrocytic 1	*SPTAN1*	0.613	0.024
Golgi-associated plant pathogenesis-related protein 1	*GLIPR2*	0.638	0.013
*N*-acetylglucosamine-6-sulfatase	*GNS*	0.667	0.021
Calnexin	*CANX*	0.675	0.028
Protein disulfide-isomerase A3	*PDIA3*	0.681	0.006
Voltage-dependent anion-selective channel protein 3	*VDAC3*	0.685	0.007
Actin, cytoplasmic 1	*ACTB*	0.690	0.013
Actin, cytoplasmic 2	*ACTG1*	0.690	0.012
Prelamin-A/C	*LMNA*	0.698	0.013
Calreticulin	*CALR*	0.740	0.020
Tubulin β-4A chain	*TUBB4A*	0.836	0.040
Tubulin β-4B chain	*TUBB4B*	0.892	0.031

**Table 3:** The table shows average fold change (FC) in protein levels upon incubation of primary human fetal retinal pigment epithelial cells (RPE) in Dulbecco’s Modified Eagle’s Medium (DMEM) with 1% sericin for seven days in comparison to control (cultured in DMEM alone). FC was calculated as follows: FC = ((mean of protein expressions in RPE cultured in DMEM with 1% sericin)/(mean of protein expressions in RPE cultured in DMEM without sericin)). Data are averages from triplicates. CYR61 = Cysteine-rich angiogenic inducer 61 protein.

Protein Name	MS/MS		Immunofluorescence		Immunoblotting	
	FC	*p*-Value	FC	*p*-Value	FC	*p*-Value
CYR61	7.3	0.02	1.10	0.17	1.05	0.90
Laminin subunit β-2	10.5	0.001	1.91	0.007	1.11	0.43
Superoxide dismutase	5.4	0.003	1.22	0.25	1.36	0.23

## Supplementary Materials

Supplementary Materials are available online. Based on the differentially expressed proteins, Ingenuity Pathway Analysis (IPA) predicted that the NF-κB pathway was activated in primary human fetal retinal pigment epithelial cells (RPE) cultured in Dulbecco’s Modified Eagle’s Medium (DMEM) with 1% sericin. Activation of the NF-κB pathway was indicated by upregulation of several of its regulator proteins and the concurrent downregulation of proteins that are known to be inhibited by the NF-κB pathway. The entries in the table show how a target (row) supports the prediction of a regulator (column).
